# Antimicrobial activity of dietary supplements based on bacterial lysate of *Lactobacillus rhamnosus* DV

**DOI:** 10.3389/fcimb.2023.1211952

**Published:** 2023-08-25

**Authors:** Yurii Penchuk, Maryana Savytska, Nazarii Kobyliak, Danylo Ostapchenko, Igor Kolodiy, Svitlana Onysenko, Olena Tsyryuk, Oleksandr Korotkyi, Fedir Grygoriev, Tetyana Falalyeyeva

**Affiliations:** ^1^ Educational and Scientific Centre, Institute of Biology and Medicine, Taras Shevchenko National University of Kyiv, Kyiv, Ukraine; ^2^ Department of Normal Physiology Danylo Halytsky Lviv National Medical University, Lviv, Ukraine; ^3^ Endocrinology Department, Bogomolets National Medical University, Kyiv, Ukraine; ^4^ Scientific Department, Medical Laboratory CSD, Kyiv, Ukraine; ^5^ Department of Biotechnology and Microbiology, National University of Food Technologies, Kyiv, Ukraine; ^6^ Scientific Department, MirImmunoPharm LLC, Kyiv, Ukraine

**Keywords:** bacterial lysate, postbiotics, metabiotics, lactic acid bacteria, antimicrobial activity, plant extracts

## Abstract

**Introduction:**

According to WHO, antibiotic resistance is increasing to hazardous levels worldwide. Candidiasis often occurs after taking antibiotics. Therefore, antibiotic resistance is a global problem and searching for antibacterial agents is necessary.

**Aim:**

To determine the antimicrobial activity of bacterial lysate of *Lactobacillus (L.) rhamnosus* DV separately and with plant extracts against bacterial and yeast test cultures.

**Material and methods:**

Antimicrobial activity of Del-Immune V^®^ (cell wall and DNA fragments from a *L. rhamnosus* DV) separately and with cinnamon, beetroot, and blackcurrant extracts was determined by the minimum inhibitory concentration (MIC). Twofold serial dilutions determined the MIC in previously prepared meat-peptone broth (MPB) for bacteria and liquid wort for yeast. In the study, gram-negative (*Escherichia coli* IEM-1, *Proteus vulgaris* PА-12, *Pseudomonas* sp. MI-2, *L. rhamnosus* 13/2) and gram-positive (*Bacillus (B.) subtilis* BТ-2, *Staphylococcus aureus* BМС-1) bacteria, as well as yeast (*Candida (C.) albicans* D-6, *C. tropicalis* PE-2, *C. utilis* BVS-65) were used as test cultures.

**Results:**

The MIC for the studied bacterial test cultures after application of *L. rhamnosus* DV bacterial lysates was from 1.0 ± 0.05 mg/mL to 12.5 ± 0.63 mg/mL, which was significantly less than that of the thermally inactivated control (MIC from 125.0 ± 6.25 mg/mL to 250.0 ± 12.5 mg/mL). *B. subtilis* BT-2 culture was the least sensitive to the action of the bacterial lysate (MIC—12.5 ± 0.63 mg/mL). It showed the best antibacterial and antifungal effect bacterial lysate with the phytonutrient blackcurrant.

**Conclusions:**

It was demonstrated that bacterial lysate of lactic acid bacteria *L. rhamnosus* DV exhibits antibacterial and antifungal properties during direct contact with pathogenic agents.

## Introduction

The scientific literature points to the beneficial properties of probiotics in the process of regulating metabolism (Falalyeyeva et al., 2022; Kobyliak et al., 2022; Falalyeyeva et al., 2023; Lazarenko et al., 2023), yet at the same time, some scientific papers question the effectiveness and safety of probiotics (Merenstein et al., 2023). In turn, postbiotics and metabiotics are preparations of inanimate microorganisms and/or their components, which are directly identified with the safety of their use and the health benefits of the host (Bourebaba et al., 2022). Due to the chemical structure of postbiotics and metabiotics, it is found that they have many health benefits; in particular, they have a local effect on certain tissues of the intestinal epithelium and influence on many other organs and tissues. Postbiotics metabolites and metabiotics structural cell fragments create the appearance of a therapeutic effect of probiotics, which, in turn, limits the risk of introducing living microorganisms into a weakened immune defense. It should also be pointed out that postbiotics and metabiotics are more stable and have a longer shelf life. Inactivation of bacterial cells can be achieved by physical means (mechanical destruction, high hydrostatic pressure, γ or UV irradiation, heat treatment, freeze drying, ultrasonication) or chemical methods (acid deactivation) (De Almada et al., 2016).

In the current scientific discussion, the term “postbiotic” also comprises cell-free supernatants with bacterial metabolites. These include soluble factors (products or by-products of metabolism) released by living bacteria or released after bacterial lysis. The term “metabiotics” is often used for determining probiotic cell wall fragments and DNA motifs after probiotic cell enzymatic destruction (Oberg et al., 2011; Cicenia et al., 2014). Such soluble factors have been isolated from a number of strains of bacteria. They are short-chain fatty acids (SCFA), peptides, enzymes, teichoic acids, muropeptides derived from peptidoglycans, endo- and exopolysaccharides, cell surface proteins, plasmalogens, vitamins, and organic acids (Chen and Hoover, 2003; Cavallari et al., 2017). Nisin, a widely used preservative in several foods (dairy products, infant formula, canned soups), can be produced by *Lactococcus lactis* subsp. lactis (Tomasik and Tomasik, 2020).

Immune function and alleviation of digestive problems is also the relevant area of use of postbiotics today. According to some studies, postbiotic supplements can help with irritable bowel syndrome (IBS) and the symptoms of inflammatory bowel disease (IBD), as well as prevent respiratory tract infections (Thorakkattu et al., 2022). Although the exact processes are not yet well understood or unknown, *in vitro* studies have shown that postbiotics exhibit antibacterial, anti-inflammatory, immunomodulatory, antiproliferative, and antioxidant effects (Cuevas-González et al., 2020; Thorakkattu et al., 2022). The use of postbiotics as functional food has several advantages in the industrial processing and commercialization of food products. This contributes to the growth of the functional food market. From a practical standpoint, postbiotics are more stable and safer for food and pharmaceutical purposes than the living bacteria from which they are produced, because their viability is not actually required for large-scale production or consumption (Barros et al., 2020).

It is worth noting that changing the processing conditions of microbial suspensions or scaling can lead to significant structural modifications and variations in the physiological functions of postbiotics, and this will complicate the production of postbiotics on a large scale. Previously obtained results demonstrated no side effects, therapeutic potency, or probiotic lysates’ bifidogenic activity, indicating a more intense activation of endogenous immune protective factors following postbiotics. The probiotic lysates classified as postbiotics demonstrate indisputable advantages for their use as a safe and potent bio-therapeutics source with high immunotherapeutic potency involving cellular and humoral immunity (Sichel et al., 2013). Given the above, using food products as a “vehicle” for postbiotics is promising and relevant today, although it has quite a few unexplored problems.

According to the World Health Organization, antibiotic resistance is rising to dangerously high levels in all parts of the world (World Health Organization, 2020). New resistance mechanisms are emerging and spreading globally, threatening our ability to treat common infectious diseases. Candidiasis often occurs after taking antibiotics. Therefore, searching for new antimicrobial agents of natural origin is an extraordinary global problem. The work aimed to determine the antimicrobial activity of bacterial lysate of *L. rhamnosus* DV separately and with plant extracts against bacterial and yeast test cultures.

## Material and methods

### Research object

In research, the cell lysates of lactic acid bacterial strain *L. rhamnosus* DV were investigated, which were included in the Del-Immune V^®^ dietary supplement produced by MirImmunoPharm LLC (Ukraine) in cooperation with Stellar Biotics, LLC (USA).

*Content*: *L. rhamnosus* DV lysate lyophilized powder: protein 100 mg/g; DNA 118 mg/g; muramyl peptides 15.0–18.0 mg/g; pH of 1% suspension 6.5–7.5; moisture (%), no more 5.0. The dietary supplement was represented by cell lysates of lactic acid bacterial strain *L. rhamnosus* DV in capsules containing lyophilisate of cells.

Also, we checked bacterial lysate (lyophilized enzymatic lysate of cells of the lactic acid bacterial strain *Lactobacillus rhamnosus* DV—50.0 mg (mg)) with cinnamon (cinnamon extract (5:1)—200 mg (mg)), beetroot (beet root extract (10:1)—500 mg (mg)), and blackcurrant (blackcurrant extract (10:1)—200 mg (mg)) extracts. Samples of thermally treated substances devoid of biological activity were used as a control.

### Preparation of test samples

In studies, samples containing cell lysates of the lactic acid bacterial strain *L. rhamnosus* DV of various concentrations were used as postbiotics. For this, a sample of the postbiotic in 25 mg was dissolved in a sterile phosphate buffer (0.1 M, pH 7.0) to an initial volume of 25 mL. Dilution was done with the same buffer to the required concentration.

### Test culture

Bacterial strains (*Escherichia (E.) coli* IEM-1, *Staphylococcus aureus* BMS-1, *Bacillus (B.) subtilis* BT-2, *Proteus (P.) vulgaris* PA-12, *Pseudomonas* sp. MI-2, *L. rhamnosus* 13/2) and yeast (*Candida (C.) albicans* D-6, *C. tropicalis* PE-2, *C. utilis* BVS-65) from the collection of live cultures of the Department of Biotechnology and Microbiology of the National University of Food Technologies (Ukraine) were used as test cultures in determining the antimicrobial and antifungal activity of the postbiotic.

### The procedure of study design

The antimicrobial activity of the postbiotics samples was analyzed according to the minimum inhibitory concentration (MIC) (Pirog et al., 2005; GoneliMali et al., 2018). The MIC was determined by twofold serial dilutions in meat-peptone broth (MPB) for bacteria and in liquid wort for yeast. Under sterile conditions, 1 mL of the medium was introduced into 10 test tubes, 1 mL of the sample solution of the maximum concentration was added to the first one, after which it was stirred, and 1 mL was taken and transferred to the next test tube. Dilution was similarly carried out for the following 12 test tubes. From the last test tube, 1 mL of solution was taken. Thus, the final volume of each tube was 1 mL (MPB or wort and postbiotic sample solution), and the concentration of the postbiotic in each subsequent tube was reduced by half. As a control, 1 mL of the postbiotic sample was used after sterilization in an autoclave (Р = 0.05, MPa = 30 min). Next, 0.1 mL of test culture suspension (10^5^–10^6^ CFU/mL) was added to each of the test tubes and stirred. The tubes were incubated for 24 h at 28°C–30°C for bacteria and 24°C–26°C for yeast. The results were evaluated by the optical density in percentage. The MIC was considered the lowest concentration inhibiting the respective microorganisms’ growth. All assays were performed in triplicate (GoneliMali et al., 2018).

The antimicrobial activity of *L. rhamnosus* 13/2 was investigated by the methods “paper disks,” “agar blocks,” and “well” methods (Hong-Thao et al., 2016). The dosage of the live culture is in CFU/mL. The results were registered visually according to the presence/absence of growth retardation zones of the test cultures on dense nutrient media.

### Statistical analysis

Statistical processing of the experimental data was carried out as previously described (Chebbi et al., 2017). The statistical analysis was performed using STATISTICA software (Version 13.3). Data were presented in simple measures of frequency, percentage, mean, standard deviation, and range (minimum–maximum values). The significance of the difference was tested using Student’s t-test for the difference between two independent means. A p-value equal to or less than 0.05 was considered statistically significant.

## Results

The data presented in **Table 1** indicate that the MIC for the studied bacterial test cultures after application of *L. rhamnosus* DV bacterial lysates was from 1.0 ± 0.05 to 12.5 ± 0.63 mg/mL, which was significantly less than that of the thermally inactivated control (MIC from 125.0 ± 6.25 to 250.0 ± 12.5 mg/mL). It should be noted that the cell lysate of the lactic acid bacterial strain *L. rhamnosus* DV exhibited a high antimicrobial effect not only against gram-positive (*B. subtilis* BТ-2, *S. aureus* BMS-1) but also against gram-negative (*E. coli* IEM-1, *P. vulgaris* PА-12, *Pseudomonas* sp. МІ-2) bacteria. The culture of *B. subtilis* BT-2 appeared to be the least sensitive to the postbiotic (MIC—12.5 ± 0.63 mg/mL). The MIC ranged from 1.0 ± 0.05 to 4.07 ± 0.2 mg/mL for other cultures.

**Table 1:** Antibacterial activity of bacterial lysates of lactic acid bacterial strain *L. rhamnosus* DV separately and with the addition of phytonutrient from beetroot, cinnamon, and blackcurrant.

MIC, mg/mL	*Bacillus subtilis* BТ-2 *(spores)*	*E. coli* ІЕМ-1	*Staphylococcus aureus* BМС-1	*Proteus vulgaris* PА-12	*Pseudomonas* sp. МІ-2
Control	125.0 ± 6.25 (119.25-131.25)	250.0 ± 12.5 (237,5-262.5)	250.0 ± 12.5 (237.5-262.5)	125.0 ± 6.25 (119.25-131.25)	125.0 ± 6.25 (119.25-131.25)
Bacterial lysate	12.5 ± 0.63* (11.4-13.1)	4.07 ± 0.2* (3.8-4.3)	2.0 ± 0.1* (1.9-2.1)	1.0 ± 0.05* (0.95-1.05)	1.0 ± 0.05* (0.95-1.05)
Bacterial lysate with beetroot extract	16.3 ± 0.82* (15.5-17.1)	8.15 ± 0.41* (7.7-8,5)	8.15 ± 0.41* (7.7-8.5)	1.0 ± 0.05* (0.95-1.05)	2.0 ± 0.1* (1.9-2.1)
Bacterial lysate with cinnamon extract	16.3 ± 0.82* (15.5-17.1)	4.07 ± 0,2* (3.8-4.3)	4.07 ± 0.2* (3,8-4.3)	2.0 ± 0.1* (1.9-2.1)	2.0 ± 0.1* (1.9-2.1)
Bacterial lysate with blackcurrant extract	8.15 ± 0.41* (7.7-8.5)	2.0 ± 0.1* (1.9-2.1)	2.0 ± 0.1* (1.9-2.1)	1.0 ± 0.05* (0.95-1.05)	1.0 ± 0.05* (0.95-1.05)

Significant difference between two independent means using Student’s t-test at the 0.05 level (*p < 0.05 compared with control).

-Data were presented as mean ± SD (range).

It has also been shown that supplements that add plant extracts showed different antibacterial activities. The *L. rhamnosus* DV bacterial lysates with the phytonutrients beetroot and cinnamon had less antibacterial activity than a supplement without additives. It may be due to the extracts’ protective substances for bacterial test strains. Comparing these compositions showed the best antimicrobial effect of the bacterial lysates *L. rhamnosus* DV with the phytonutrient blackcurrant.

The study of the antifungal action showed that the bacterial lysates *L. rhamnosus* DV are characterized by a pronounced fungicidal activity against *Candida* yeast. Data on the study of the action of antifungal substances of bacterial lysates of various postbiotics are presented in **Table 2**.

**Table 2:** Antifungal activity of bacterial lysates of lactic acid bacterial strain *L. rhamnosus* DV separately and with the addition of phytonutrient from beetroot, cinnamon, and blackcurrant.

MIC, mg/mL	*Candida tropicalis* PE-2	*Candida albicans* D-6	*Candida utilis* BVS-65
Control	500.0 ± 25 (475-525)	500.0 ± 25 (475-525)	500.0 ± 25 (475-525)
Bacterial lysate	125.0 ± 6.25* (119.25-131.25)	65.2 ± 3.25* (63-68.5)	125.0 ± 6.25* (119.25-131.25)
Bacterial lysate with beetroot extract	125.0 ± 6.25* (119.25-131.25)	65.2 ± 3.25* (63-68.5)	125.0 ± 6.25* (119.25-131.25)
Bacterial lysate with cinnamon extract	65.2 ± 3.25* (63-68.5)	32.6 ± 1.63* (31-34.2)	65.2 ± 3.25* (63-68.5)
Bacterial lysate with blackcurrant extract	65.2 ± 3.25* (63-68.5)	32.6 ± 1.63* (31-34.2)	32.6 ± 1.63* (31-34.2)

Significant difference between two independent means using Student’s t-test at the 0.05 level (*p < 0.05 compared with control).

-Data were presented as mean ± SD (range).

These findings indicate that the bacterial cell lysates *L. rhamnosus* DV (with and without plant extracts) show less antifungal activity against test microorganisms than bacterial test microorganisms. Thus, the MIC was in the range of 65.2 ± 3.25 to 125.0 ± 6.25 mg/mL, which was 4 (p < 0.05) to 8 (p < 0.05) times less than the control. Despite this, we may consider these concentrations therapeutic, given the low toxicity level of the postbiotic. However, it should be noted that the supplements with extracts were more active against yeast test cultures. The bacterial cell lysates *L. rhamnosus* DV with the phytonutrient blackcurrant had more profound antifungal activity (**Table 2**).

Experiments were conducted *in vitro*, so we can talk about the direct effect of the postbiotic on microorganisms. It was necessary to compare the influence of probiotic lysate with the living cells *L. rhamnosus*. Since it is impossible to check the activity of living cells using the method specified in the article, it was decided to use the following methods: “paper disks,” “agar blocks,” and “well” methods. Dosage of the drug was carried out in mg/mL, and that in the case of live culture was in CFU/mL.

It was shown that when testing the activity by the “well method,” a live culture of *L. rhamnosus* prevents the growth of *S. aureus* BMS-1 at a dilution of 10^5^ CFU/mL. The application dose was 200 μL (2*10^4^ CFU in 200 μL). All other cultures showed resistance to the first dilution, which was at the level of 10^7^ CFU/mL. The “paper disk” and “agar block” methods showed no growth retardation of the test cultures due to the insignificant amounts of living cells in the agar block and paper disks. Because the units of measurement cannot be directly compared, we can only conclude the qualitative and not the quantitative component of antimicrobial activity in relation to the test cultures of microorganisms.

## Discussion

Impaired wound healing is a growing medical problem, and very few approved drugs with documented clinical efficacy are available. Lactic acid bacteria exhibit antibacterial activity due to the production of lactic acid, acetic acid, hydrogen peroxide, diacetyl, CO_2_, and bacteriocins. However, reports on using lactic acid bacteria in bioconservation are limited due to their antifungal effects (Maier et al., 2010). It has been suggested that topical lactic acid bacteria can improve skin health or combat disease. It has been shown that specific lactobacilli strains have a beneficial role in the wound healing process, in defense against the inflammatory processes that affect the skin, and in resistance to infections by interfering with pathogens (Baquerizo Nole et al., 2014; Lukic et al., 2017; Knackstedt et al., 2020). CXCL12-expressing lactic acid bacteria, *Limosilactobacillus reuteri* (ILP100-Topical), has been demonstrated to accelerate wound healing in controlled preclinical models (Öhnstedt et al., 2022). In the clinical study, ILP100-Topical was safe and well-tolerated in all individuals and doses with no systemic exposure. A combined cohort analysis showed a significantly more significant proportion of healed wounds by multi-dosing of ILP100-Topical when compared with placebo. The time to first registered healing was shortened by 6 days on average and by 10 days at the highest dose. ILP100-Topical increased the density of CXCL12+ cells in the wounds and local wound blood perfusion (Öhnstedt et al., 2023).

The International Scientific Association for Probiotics and Prebiotics raised questions regarding the safety of probiotics, especially among vulnerable populations (such as newborns, pregnant women, patients with short bowel syndrome, and immunocompromised). The authors noted that the most significant danger is the possibility of antibiotic resistance gene transfer via transformation, the potential impact of probiotic−induced changes in microbiomes, and drug interactions (Merenstein et al., 2023). Recent scientific studies on the stated issues point to the benefits of some postbiotics in treating metabolic disorders (Bourebaba et al., 2022). The practical use of probiotics and the study of the mechanism of their action were made lately to find that a certain level of biological activity is preserved by dead probiotic cells and even their lysates, which are the natural mixes of metabiotic and postbiotic substances—a biological activity which is strongly oriented toward gut health and immune system regulation. Because probiotic lysates demonstrated biological activity without any of the potential adverse side effects associated with live bacterial cells, one of the future goals is research of the novel postbiotics and metabiotics substances as well as their individual structures and biological characteristics for understanding their way of communications with host cells and microbiota representatives. That is why studies conducted to study the antibacterial activity of postbiotics are extremely important.

Data about using probiotic culture lysates in medical practice to control infectious diseases are not widely presented. It has also been shown that *L. rhamnosus* LR lysate improves skin barrier function in a reconstructed human epidermis model (Jung et al., 2019). In our study, supplements containing bacterial lysates of lactic acid bacterial strain *L. rhamnosus* DV exhibit antibacterial and antifungal activity. The spectrum of antimicrobial activity of the studied medications concerned gram-positive and gram-negative bacteria, along with yeast-like fungi of the genus Candida. Antimicrobial activity was demonstrated by bacterial lysate samples that were thermally inactivated, indicating that the active substances are thermolabile.

It is widely accepted that bioactive compounds extracted from plants significantly impact human health. As a result, it is crucial to comprehend the antimicrobial effectiveness and mode of operation of these plant-derived bioactive compounds (Kha and Le, 2021). Plants with medicinal properties contain various phytochemicals like flavonoids, alkaloids, tannins, and terpenoids. These natural compounds have antimicrobial and antioxidant properties (GoneliMali et al., 2018). Many plant species have been extensively studied for their antimicrobial properties. Cinnamon, beetroot, blackcurrant, garlic, basil, curry, ginger, sage, mustard, and other herbs have crude extracts that exhibit antimicrobial effects against a wide range of Gram-positive and Gram-negative bacteria (Alzoreky and Nakahara, 2003; Castro et al., 2008; Ikuta et al., 2012; Suriyaprom et al., 2022). In this study, the bacterial lysate added with plant extracts (beetroot and cinnamon) exhibited less antimicrobial activity against bacterial test cultures, which may be associated with the protective properties of the extract components. However, in general, minimal inhibitory doses can also be considered therapeutic. It showed the best antimicrobial effect bacterial lysates of lactic acid bacterial strain *L. rhamnosus* DV with the phytonutrient blackcurrant. Probiotic lysate showed high activity against gram-positive and gram-negative bacteria and Candida yeasts. Instead, the live culture *L. rhamnosus* showed an inhibitory effect only of a gram-positive bacterium—*S. aureus* BMS-1.

Jung et al. (2019) noted that it would be interesting to examine whether the application of live *L. rhamnosus* can manifest skin barrier-protective effects. However, cosmetics must not be contaminated with microorganisms including probiotic species. The FDA regulates that acceptable limits for total (not pathogenic) microorganisms in cosmetics are 500 colony forming units (CFU) per gram in eye-area products and 1,000 CFU/g for other area products (Herrera, [[NoYear]]). Therefore, using live probiotic strains in cosmetic products must overcome this hurdle whereas different biotherapeutic approaches beyond cosmetics are ongoing with live probiotics (Jung et al., 2019). Indeed, in our work, we have shown that a live culture of *L. rhamnosus* 13/2 prevents the growth of *S. aureus* BMS-1 at a huge concentration—10^5^ CFU/mL. All other cultures showed resistance to the first dilution, which was at the level of 10^7^ CFU/mL. Therefore, the obtained data indicate the ineffectiveness of a live culture of *L. rhamnosus* 13/2 in inhibiting the growth of test cultures of microorganisms.

Given the above, bacterial lysate, which refers to postbiotics, can present the possibility of developing new and more effective therapeutic strategies with a more advanced safety profile while circumventing the risks related to the use of living microorganisms.

## Conclusion

Therefore, it was demonstrated that bacterial lysate of lactic acid bacteria *L. rhamnosus* DV exhibits antibacterial and antifungal properties during direct contact with pathogenic agents. The bacterial lysate of lactic acid bacteria *L. rhamnosus* DV showed the best antibacterial and antifungal effects of bacterial lysate with the phytonutrient blackcurrant. Obtained results opened a new area for bacterial lysates of lactic acid bacterial strain *L. rhamnosus* DV topical application, combined with modulation of innate skin immunity with direct impact on infectious agents.

## Data availability statement

The raw data supporting the conclusions of this article will be made available by the authors, without undue reservation.

## Author contributions

YP, MS, TF, and NK contributed to the conceptualization and the original idea of this manuscript. IK and DO contributed to methodology and reviewed the literature. SO, OT, and OK were involved in validation and revised and validated the literature findings. TF and YP performed formal analysis. YP and IK contributed to investigation. TF and NK were involved in data curation. NK, YP, MS, and TF did the writing—original draft preparation. TF, YP, and MS did the writing—review and editing. NK did visualization. TF and NK were involved in supervision. TF, NK, FG, and YP contributed to project administration. All authors contributed to the article and approved the submitted version.
